# Association of Plasma Adiponectin and Oxidized Low-Density Lipoprotein with Carotid Intima-Media Thickness in Diabetic Nephropathy

**DOI:** 10.1155/2015/507265

**Published:** 2015-05-12

**Authors:** Anna Tavridou, Anastasia Georgoulidou, Athanasios Roumeliotis, Stefanos Roumeliotis, Efstathia Giannakopoulou, Nikolaos Papanas, Ploumis Passadakis, Vangelis G. Manolopoulos, Vassilis Vargemezis

**Affiliations:** ^1^Laboratory of Pharmacology, Medical School, Democritus University of Thrace, 68100 Alexandroupolis, Greece; ^2^Department of Nephrology, Medical School, Democritus University of Thrace, 68100 Alexandroupolis, Greece; ^3^Second Department of Internal Medicine, Diabetes Clinic, Medical School, Democritus University of Thrace, 68100 Alexandroupolis, Greece

## Abstract

*Aims*. We sought to determine the association between levels of adiponectin and oxidized low-density lipoprotein (ox-LDL) in patients with diabetic nephropathy as well as their effect on carotid intima-media thickness (cIMT). *Methods*. Adiponectin and ox-LDL were determined in 25 diabetic patients without nephropathy and 94 patients at different stages of diabetic nephropathy including subjects on hemodialysis. cIMT was measured using real-time B-mode ultrasonography. *Results*. Plasma adiponectin levels increased significantly with severity of diabetic nephropathy (*P* = 0.002), on the contrary to ox-LDL which decreased with disease severity (*P* < 0.001). cIMT was significantly higher at late stages of diabetic nephropathy compared with early stages (*P* = 0.022). Adiponectin was a significant negative predictor of ox-LDL levels (*β* = −5.45, *P* = 0.023), independently of confounding factors. There was no significant correlation between cIMT and adiponectin or ox-LDL either in the total sample population or according to disease staging. Cluster analysis showed that patients with the highest cIMT values, highest levels of adiponectin, and lowest levels of ox-LDL were included in one cluster and all assigned to stage 5 of diabetic nephropathy. *Conclusions*. There was no significant association between adiponectin or ox-LDL and cIMT and, therefore, other factors affecting this surrogate marker of cardiovascular disease in diabetic nephropathy should be sought.

## 1. Introduction

Diabetic nephropathy is a complex disease affecting approximately 30% of patients with type 2 diabetes mellitus (T2DM) [1] and leading to increased morbidity and mortality. The pathogenesis of diabetic nephropathy is multifactorial; however, the mechanisms that drive its development remain largely undetermined [2, 3]. During the course of diabetic nephropathy, the functional impairment and structural remodeling of the kidney, induced by hyperglycemic injury, are linked to changes in several cellular events and activation of signaling pathways.

Adipokines, including adiponectin, exert regulatory activities on various target tissues. Most clinical and experimental studies support a controversial role of adiponectin in diabetic nephropathy. Adiponectin-knockout (Ad^−/−^) mice exhibited increased albuminuria and fusion of podocyte foot processes, whereas infusion of adiponectin reduced oxidative stress and reversed the albuminuria [4]. However, the beneficial roles of adiponectin may not be completely applied to the entire course of diabetic nephropathy. In early diabetic nephropathy, endothelial dysfunction is associated with low circulating adiponectin [5]. Nevertheless, in advanced diabetic nephropathy with macroalbuminuria or renal insufficiency, increased serum adiponectin levels were observed [6–8], predicting coexisting vascular endothelial dysfunction [6]. In addition, high serum adiponectin levels predict mortality and progression to end stage renal disease in type I diabetic patients [9]. The discrepancy between beneficial effects of adiponectin, observed mainly in early diabetic nephropathy, and association of adiponectin with negative prognosis in diabetic and nondiabetic patients with more advanced chronic kidney disease (CKD) is generally explained by the confounding effect of inflammation of CKD and dialysis which at the same time impacts on prognosis and triggers increased adiponectin synthesis [10, 11]. Moreover, the expected inverse relationship between adiponectin and clinical outcomes may be modified by differential retention of high-molecular weight forms of adiponectin in renal failure, the nutritional and inflammatory status, adiponectin gene polymorphisms, and combination of these factors [12].

The role of lipid abnormalities in the pathogenesis of glomerular injury has been demonstrated in animal models in addition to a link between hypercholesterolemia and diabetic nephropathy in humans [13]. Localized tissue oxidative stress plays a key role in the development of diabetic nephropathy [14] and oxidized low-density lipoprotein (ox-LDL) is induced in the onset of the disease [15]. Elevated plasma ox-LDL levels were observed in T2DM patients with macroalbuminuria [16], whereas significantly lower levels of ox-LDL were observed in CKD patients undergoing hemodialysis compared with controls [17]. Decreased adiponectin levels are an indicator of increased oxidative state in the arterial wall, whereas adiponectin treatment markedly suppresses foam cell formation in ox-LDL-treated macrophages from diabetic subjects [18]. An inverse association between adiponectin and ox-LDL levels was found in T2DM patients with coronary artery disease [19] as well as in hemodialysis patients [17]. However, the interplay between adiponectin and ox-LDL levels has not been studied in patients at different stages of diabetic nephropathy.

Although circulating adiponectin has been suggested as a possible marker of cardiovascular disease in the general population, it is a poor predictor of cardiovascular risk in both the CKD and renal transplant population [20]. Noninvasive techniques are widely used to estimate the stage of atherosclerotic changes and carotid intima-media thickness (cIMT) is a useful marker of diabetic macroangiopathy as well as the extent of coronary artery disease in patients with impaired renal function [21]. Findings on the association between adiponectin levels and cIMT in kidney disease are contradictory. In predialysis CKD patients, an inverse association or no association was shown [22, 23]. Similarly, in end stage renal disease, adiponectin was shown either to correlate inversely with cIMT [24] or not to be associated with cIMT [25]. On the contrary, a positive correlation between cIMT and adiponectin has been reported in patients with diabetic nephropathy [6].

In the present study, we sought to explore (a) the association between circulating levels of adiponectin and ox-LDL in patients with diabetic nephropathy and (b) their association with cIMT in the studied subjects.

## 2. Methods

### 2.1. Subjects

A total of 119 unrelated patients of Caucasian origin were included in the study. The criteria for determining subjects with T2DM for inclusion in the study have been described before [26]. All eligible patients who consented to participate in the study had to have a documented history of type 2 diabetes for at least 10 years.

Established diabetic nephropathy was defined by microalbuminuria (30–300 mg/g creatinine) or persistent albuminuria (>300 mg/g creatinine) in two out of three consecutive measurements in sterile spot urine sample during a 6-month period, presence of diabetic retinopathy, and absence of other kidney or urinary tract disease [27, 28]. Diabetic retinopathy (DR) was included in the eligibility criteria for this study, since it is frequent in the presence of diabetic nephropathy and is a clue for its diagnosis. DR was assessed by fundoscopy, after pupillary dilatation. The ophthalmologic exam result was classified as normal, nonproliferative, and proliferative retinopathy. Patients were considered to have DR if they showed nonproliferative or proliferative stage. Alternatively, the patients might have a history of retinal laser surgery (photocoagulation) for DR. The diagnosis and classification of CKD stages were established according to the criteria from the Clinical Practice Guidelines for Chronic Kidney Disease from the National Kidney Foundation—Kidney Disease Outcomes Quality Initiative [29]. Absence of diabetic nephropathy (controls) was defined as absence of diabetic retinopathy, eGFR above 60 mL/min, and persistent normoalbuminuria (0–30 mg/g creatinine) after at least 10 years of type 2 diabetes. Patients with clinical or laboratory evidence of nondiabetic nephropathy or urinary tract disease were excluded from the study. All stage 5 CKD patients had been under regular hemodialysis, for at least 6 months, and were dialyzed thrice weekly for 4 hours per session (CKD-5D).

All diabetic patients were regular patients at the Diabetes Clinic and Nephrology Clinic of Academic General Hospital of Alexandroupolis (Greece) and gave written informed consent. The study was approved by the Ethics Committee of the Scientific Council of the University General Hospital of Alexandroupolis and was in accordance with Helsinki Declaration of Human Rights.

### 2.2. Laboratory Methods

Fasting blood was obtained from all patients and plasma was stored at −20°C until analysis. Blood samples were collected from hemodialysis patients after an overnight fasting of 8 h, immediately before the start of a routine 4 h hemodialysis session, as described in previous studies [17, 30]. Blood was drawn from all patients into EDTA-containing tubes and into tubes without anticoagulant in order to obtain plasma, whole blood, and serum. Samples for fasting blood glucose levels, HbA1c, total cholesterol, low-density lipoprotein cholesterol (LDL-cholesterol), high-density lipoprotein cholesterol (HDL-cholesterol), triglycerides, and creatinine were transferred to the laboratory and assayed immediately. For adiponectin and ox-LDL, the samples were centrifuged immediately and plasma was stored at −20°C until analysis.

According to guidelines of American clinical practice (K/DOQI), the five stages of renal insufficiency based on eGFR are as follows: I ≥ 90, II = 60–89, III = 30–59, IV = 15–29, and V < 15 [9, 31]. The glomerular filtration rate (GFR) was estimated (eGFR) using the chronic kidney disease epidemiology collaboration (CKD-EPI) equation, which is more accurate and less biased than the MDRD Study equation, especially in patients with higher GFR, resulting in reduced misclassification of CKD [32].

Plasma concentrations of total adiponectin and ox-LDL were quantitated by enzyme-linked immunosorbent assay (ELISA) according to the manufacturer's instructions (human adiponectin ELISA kit, Buhlmann, Switzerland; human ox-LDL ELISA kit, Mercodia, Sweden). According to the manufacturers, the detection limits for adiponectin and ox-LDL assays were 0.08 ng/mL and 0.3 U/L, respectively. Intra- and interassay coefficients of variation were <15% and <10% for adiponectin and ox-LDL, respectively.

### 2.3. Measurement of cIMT

Measurements of cIMT were obtained from 117 out of 124 patients with diabetic nephropathy recruited in this study. cIMT was considered the distance from the leading edge of the lumen-intima interface to the leading edge of the intima-adventitia interface. Measurements of cIMT were performed online by a single trained sonographer, using a high resolution, real-time ultrasonograph with a 7,5 MHz transducer (ATL Ultrasound HDI 1300, Philips, Bothell, WA, USA). Three measurements were performed for each of the common carotid arteries at 0.5, 1, and 1.5 cm distance from carotid bulb and mean cIMT was calculated by averaging the six measurements. Maximum cIMT was defined as the greatest value of those measurement points of both sides.

### 2.4. Statistical Analysis

Statistical analyses were performed using the Statistical Package for Social Sciences (SPSS 18.0 for Windows). Data for patients with diabetic nephropathy at stages 3 and 4 were merged for analysis due to the small number of subjects at stage 4 of the disease. Data were tested for normality by Kolmogorov-Smirnov test. Normally distributed continuous variables are presented as mean (S.D.), whereas nonnormally distributed continuous variables are presented as median (range). Comparisons for normally distributed continuous variables among stages of diabetic nephropathy were performed with one-way ANOVA. Nonnormally distributed continuous variables were compared by Mann-Whitney test or Kruskal-Wallis test as appropriate, whereas categorical variables were compared by *χ* ^2^ test. Bivariate associations between variables were examined using Spearman's correlation coefficient. Correlation analysis of cIMT with various factors (age, sex, body mass index (BMI), systolic blood pressure, smoking, eGFR, LDL-cholesterol, HDL-cholesterol, duration of T2DM, and statin treatment) was conducted on an exploratory basis to determine possible predictors of cIMT. Stepwise multiple regression analysis was performed to study the relationship between ox-LDL and adiponectin, adjusting for confounding variables (LDL-cholesterol, triglycerides, and eGFR). The same type of analysis was used to assess the significance of possible predictors of cIMT (log-transformed) as determined by correlation analysis. Cluster analysis methods lead to the definition of groups with similar values of certain attributes such as laboratory variables (qualitative or quantitative analytes) or other variables (e.g., anthropometric) [33]. Using a matrix of distance measurements, cluster analysis finds groups of subjects more similar to each other than to those in other groups. The SPSS TwoStep cluster analysis is an algorithm that finds the optimal number of clusters using both continuous and categorical variables. In the present model, age, fasting glucose, adiponectin, and ox-LDL were included as continuous variables, whereas stage of diabetic nephropathy was included as categorical variable. Differences were considered statistically significant at a *P* value of <0.05.

## 3. Results

Anthropometric, biochemical, and clinical data of patients with diabetic nephropathy in total and according to stages of disease are presented in Table 1. There was no significant difference in age and the proportion of sexes across categories of diabetic nephropathy severity. The duration of T2DM was significantly different across groups and increased in late stages of diabetic nephropathy (*P* = 0.01). Although total cholesterol and LDL-cholesterol did not differ significantly among groups, HDL-cholesterol decreased whereas triglycerides increased with disease progression (*P* < 0.001 and *P* = 0.006, resp.). Plasma adiponectin increased significantly with disease severity (*P* = 0.002). Post hoc analysis showed significant differences between control subjects and subjects with stage 5 diabetic nephropathy (*P* = 0.002). Moreover, significant differences were observed between subjects with (a) stages 1/2 and 3/4 (*P* = 0.027), (b) stages 1/2 and 5 (*P* = 0.001), and (c) stages 3/4 and 5 (*P* = 0.048). Conversely, circulating ox-LDL decreased significantly with disease severity (*P* < 0.001). Post hoc analysis showed that this was due to a difference between control subjects and subjects with stage 5 diabetic nephropathy (*P* = 0.002) as well as between subjects with stage 3/4 and stage 5 (*P* = 0.001). In addition, the ratio of ox-LDL/LDL-cholesterol (U/mg) did not differ among groups (results not shown). We also investigated the effect of angiotensin-converting enzyme inhibitors, angiotensin receptor blockers, metformin, and statins on adiponectin and ox-LDL levels. The only difference identified for adiponectin was that patients on metformin (*N* = 35) had significantly lower levels of adiponectin compared with those not taking the drug (6.1 versus 9.2 *μ*g/mL, *P* = 0.001). Moreover, there was an effect of statins on ox-LDL levels, as these were significantly lower in patients (*N* = 95) treated with statins compared with levels in untreated patients (59.1 versus 68.9 U/L, *P* = 0.04). Finally, cIMT was significantly higher at late stages of diabetic nephropathy compared with control subjects (*P* = 0.022). The latter had significantly lower cIMT values compared with patients at stage 3/4 or 5 (*P* = 0.009 and *P* = 0.017, resp.).

Table 2 shows the correlation matrix between adiponectin and ox-LDL with several characteristics of the patients studied. There was a significant inverse association between adiponectin and ox-LDL in the total sample population (*r* = −0.29, *P* < 0.01) as well as in different stages of diabetic nephropathy (results not shown). Moreover, adiponectin positively correlated with age, whereas it inversely correlated with eGFR and BMI. However, there was no significant correlation between adiponectin and glucose or lipids. A significant positive association was observed between ox-LDL and lipids/eGFR. After adjustment for age and BMI, plasma adiponectin positively correlated with stages of diabetic nephropathy (*r* = 0.23, *P* = 0.014). Also, after adjustment for LDL-cholesterol and triglycerides, ox-LDL was inversely correlated with stages of diabetic nephropathy (*r* = −0.33, *P* < 0.001). In multiple regression analysis (Table 3) including the variables which correlated with ox-LDL (adiponectin, eGFR, LDL-cholesterol, and triglycerides), adiponectin was a significant negative predictor of ox-LDL levels (*β* = −5.02, *P* = 0.049), independently of LDL-cholesterol, triglycerides, and eGFR.

There was no significant correlation between cIMT and adiponectin or ox-LDL either in the total sample population (*r* = 0.14, *P* = 0.13 and *r* = −0.003, *P* = 0.97, resp.) or in any stage of diabetic nephropathy (results not shown). cIMT significantly correlated with age (*r* = 0.24, *P* = 0.008), sex (*r* = −0.32, *P* = 0.001), eGFR (*r* = −0.23, *P* = 0.004), and HDL-cholesterol (*r* = −0.18, *P* = 0.049). Also, cIMT was significantly lower in patients treated with statins compared with untreated patients (0.92 mm versus 1.00 mm, *P* = 0.04). In multiple regression analysis including the above variables (Table 3), the only independent predictors of cIMT were eGFR (*β* = −0.002, *P* = 0.003) and sex (*β* = −0.102, *P* = 0.017).

**Figure 1:**
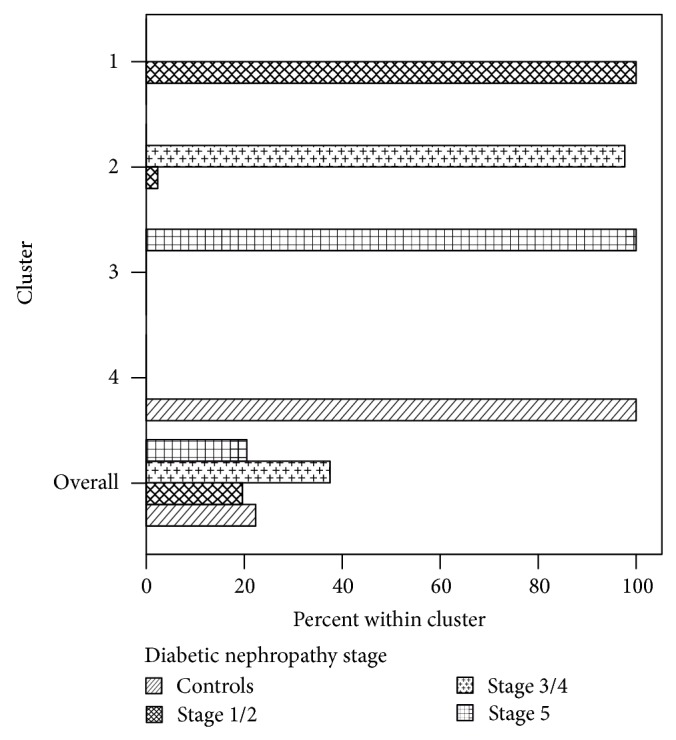
Within cluster percent of patients according to the stage of diabetic nephropathy.

Cluster analysis was performed to further explore the interrelationship of adiponectin, ox-LDL, and cIMT regarding the stage of diabetic nephropathy (Table 4). Based on four variables entered in the model (adiponectin, ox-LDL, cIMT, and age), four clusters were formed, each one distinctively characterizing each stage of diabetic nephropathy. Patients with the highest cIMT values, highest levels of adiponectin, and lowest levels of ox-LDL were included in cluster 3 and all assigned to stage 5 of diabetic nephropathy. Conversely, patients with the lowest cIMT values and low levels of adiponectin were included in cluster 4 and all assigned to the control group.  Figure 1 shows within cluster percent of patients according to the stage of diabetic nephropathy.

## 4. Discussion

In patients with diabetes mellitus as well as in those with CKD, cardiovascular disease is the leading cause of mortality. The increased cardiovascular disease risk cannot solely be explained by traditional risk factors and, therefore, identification of novel modifiable risk factors is important in preventing cardiovascular disease in diabetic nephropathy.

In the present study, we assessed circulating adiponectin and ox-LDL levels in patients with diabetic nephropathy as well as cIMT as a noninvasive surrogate of atherosclerotic status. Plasma adiponectin levels increased significantly with disease severity, in agreement with previous studies [7, 8]. Moreover, adiponectin positively correlated with age and stages of diabetic nephropathy, whereas it inversely correlated with eGFR and BMI, as previously observed [7]. Several features of uremia, such as inflammation, oxidative stress, and sympathetic overactivity, may decrease adiponectin expression, whereas other features, such as decreased renal function, proteinuria, and protein energy wasting, may increase circulating adiponectin levels [34]. It is controversial whether adiponectin levels are increased in chronic kidney disease as a result of declining GFR and, therefore, decreased catabolism or in response to other uremic factors, since patients with advanced diabetic nephropathy have both increased adiponectin serum concentrations and increased urinary excretion [35]. It has been suggested that increased adiponectin levels in overt diabetic nephropathy might be a physiological response to counteract renal tubular injury preventing the further progression of diabetic nephropathy through its anti-inflammatory and antiatherogenic effects [36]. It was recently shown that renal tubular cells secrete adiponectin which increases upon inflammatory stimulus [37] and, therefore, may contribute to the increase in circulating adiponectin levels observed in diabetic nephropathy. We found that patients taking metformin have lower levels of plasma adiponectin. However, this is probably not a real effect of metformin on adiponectin levels but can be explained by the fact that this oral hypoglycemic agent is contraindicated in patients with advanced kidney disease due to the risk for lactic acidosis. The Canadian Diabetes Association practice guidelines recommend that metformin should be stopped when the patient has progressed to stage 4 CKD (GFR < 30 mL/min/1.73 m^2^) and should be used with extreme caution in stage 3 CKD patients (GFR between 30 and 59 mL/min/1.73 m^2^) [38, 39]. In the present study, the groups of patients under metformin therapy were control patients without nephropathy, patients in stages 1 and 2, and only the minority of patients (4 out of 35) in stage 3 CKD. Therefore, the difference in adiponectin levels according to metformin treatment seems to be due to confounding by its restricted administration in late stages of CKD.

Plasma ox-LDL levels were significantly decreased in patients with end stage diabetic nephropathy compared with early stages. Despite the fact that some studies have reported higher ox-LDL levels in patients undergoing hemodialysis, contradicting results have also been reported. Some authors found no difference between hemodialysis patients and controls [30, 40], whereas others described low ox-LDL levels in these patients [17, 41–43]. Possible causes of these contradictory results may be differences among study populations in age, serum lipids, or disease since most of the studies that reported elevated levels of ox-LDL in hemodialysis patients compared ox-LDL levels in nondiabetic end stage renal disease patients under hemodialysis versus healthy controls. In the present study, we assessed ox-LDL levels in diabetic patients in different stages of CKD (stages 1–4), diabetic patients under hemodialysis (stage 5), and diabetic controls without kidney failure. Although there are reports with contradictory results on several markers of oxidative stress in patients with end stage renal disease, our results are in agreement with previous studies using the same quantitation method for ox-LDL [17, 41, 42]. Since the ratio of ox-LDL/LDL-cholesterol did not differ among groups of patients, the proportion of LDL that is oxidized was similar across different stages of diabetic nephropathy. The reduction in ox-LDL levels in patients with end stage diabetic nephropathy does not necessarily imply a reduced risk of atherosclerosis. This could be due to a greater capture of ox-LDL by macrophage scavenger receptors which are increased in hemodialysis patients [42]. It is also likely that decreased ox-LDL levels in hemodialysis patients are due to a reduction in LDL particles [17]. Finally, Sevinc Ok et al. reported that ox-LDL is not associated with the progression of atherosclerosis or cardiovascular/overall mortality in hemodialysis patients [44].

It has been shown that low serum adiponectin is associated with high circulating oxidized low-density lipoprotein in patients with type 2 diabetes mellitus and coronary artery disease [19]. Similarly, we have shown an inverse association between plasma levels of adiponectin and ox-LDL in the total sample population as well as in different stages of diabetic nephropathy. In the study by Lautamäki et al. [19], adiponectin was an independent negative predictor of ox-LDL but not lipids, whereas in the study by Ribeiro et al. [17] independent associations of ox-LDL were found with lipids but not adiponectin in patients under hemodialysis. Nevertheless, in the present study, both lipids and adiponectin were independent predictors of ox-LDL. It has been recently shown that higher levels of adiponectin are associated with a more beneficial oxidative stress profile and lower levels of lipid peroxidation [45].

In patients with T2DM, increased cIMT was shown to be partly explained by lower plasma adiponectin [46]. However, in patients with diabetic nephropathy, adiponectin was shown to be associated with several indices of vascular dysfunction, including a positive correlation with cIMT, while increased plasma adiponectin was assumed to be compensatory for early vascular endothelial damage [6]. We did not detect a significant association between cIMT and adiponectin either in the total sample population or according to disease staging, probably due to differences in patient characteristics between our study and the study by Ran et al. [6]. In the latter, no patients with diabetic nephropathy on hemodialysis were included, whereas all patients were free of cardiovascular complications. Our results are in agreement with the study by von Eynatten et al. where circulating adiponectin was not a predictor of cIMT in subjects with early stage diabetic nephropathy [47]. Similarly with adiponectin, ox-LDL was not associated with cIMT in the studied population, although cIMT increased with disease staging and ox-LDL decreased. Since there was an effect of statins on cIMT, we included statin treatment in multiple regression analysis for cIMT along with age, sex, eGFR, and HDL-cholesterol. However, only sex and eGFR were significant predictors of cIMT in these patients.

Clustering techniques are used when the data are expected to group physically in various categories. Cluster analysis was used to describe the patterns of diabetic nephropathy based on the clinical, pathogenetic, and physiological features. According to this analysis, four clusters were formed, each one distinctively characterizing each stage of diabetic nephropathy. Patients with the highest cIMT values, highest levels of adiponectin, and lowest levels of ox-LDL were included in one cluster and all assigned to stage 5 of diabetic nephropathy. Definitely, the repeatability of the cluster solutions should be validated in different samples and the clinical benefit must be proven [33]. Although cluster analysis is inadequate in identifying disease causes, its results may give insights into the structure of a data set and lead to hypotheses for further investigation. In patients with end stage diabetic nephropathy, the increased atherosclerotic burden is peculiarly accompanied by a favorable profile of both adiponectin and ox-LDL. Therefore, apart from physiological and traditional factors associated with cIMT, other pathological/protective factors affecting this surrogate marker of atherosclerosis should be explored in diabetic nephropathy. For example, other modified forms of LDL as well as circulating markers of antioxidant capacity should be examined since the latter have been shown to be negative determinants of cIMT in diabetic patients [48].

To our knowledge, this is the first study assessing the interrelationship of adiponectin and ox-LDL as well as their effect on cIMT in diabetic nephropathy. However, there are certain limitations in the present study. The cross-sectional design of the study precludes establishing causality. The number of patients with diabetic nephropathy according to disease staging is small to draw definite conclusions on the associations examined. Finally, only total adiponectin was measured and not its various isoforms which could show different associations with the variables examined.

## 5. Conclusions

An inverse association between circulating adiponectin and ox-LDL was observed in diabetic nephropathy, whereas adiponectin was an independent negative predictor of ox-LDL in these patients. There was no significant association between cIMT and adiponectin or ox-LDL either in the total sample population or according to disease staging. Other factors affecting this surrogate marker of cardiovascular disease in diabetic nephropathy should be sought in prospective studies since the early detection of atherosclerotic changes using biomarkers could reduce adverse outcomes.

## Figures and Tables

**Table 1:** Anthropometric, clinical, and biochemical characteristics of patients with type 2 diabetes in the absence or presence of nephropathy at different stages. Results for continuous variables are presented as mean (S.D.) or median (range).

	Stage of diabetic nephropathy				*P* ^a^
	Control	1 & 2	3 & 4	5	
*N*	25	22	42	30	
Age (y)	64.0 (7.1)	67.5 (8.8)	69.4 (8.2)	69.1 (9.7)	0.07
Gender (M/F)	8/17	3/13	25/17	13/17	0.14
BMI (kg/m^2^)	31.9 (6.6)	30.5 (4.1)	32.3 (4.8)	29.4 (5.2)	0.10
Duration of T2DM (y)	10.8 (5.8)	13.7 (7.0)	16.9 (9.0)	16.0 (6.6)	0.01
Fasting glucose (mg/dL)	151.8 (38.1)	148.8 (43.9)	159.0 (49.2)	189.4 (99.7)	0.07
HbA1c (%)	7.4 (1.0)	7.1 (0.8)	7.8 (1.4)	7.3 (0.9)	0.10
eGFR (mL/min/1.73 m^2^)	86.2 (14.3)	77.7 (12.7)	38.4 (11.2)	7.0 (3.0)	<0.001
Total cholesterol (mg/dL)	184.8 (51.6)	174.4 (37.5)	181.1 (43.8)	159.5 (47.7)	0.15
LDL-cholesterol (mg/dL)	101.3 (42.4)	98.1 (34.5)	101.0 (33.4)	87.0 (42.0)	0.41
HDL-cholesterol (mg/dL)	48.0 (11.3)	52.6 (16.5)	45.3 (10.2)	37.5 (9.6)	<0.001
Triglycerides (mg/dL)	115.0 (52–292)	139.5 (53–320)	170.0 (59–450)	170.5 (61–551)	0.006
Plasma adiponectin (*μ*g/mL)	5.8 (2.2–12.4)	4.2 (2.4–36.5)	6.2 (2.0–38.8)	7.9 (3.2–43.2)	0.002
ox-LDL (U/L)	67.5 (21.5)	59.7 (17.3)	66.6 (17.4)	49.2 (16.3)	<0.001
Mean cIMT (mm)	0.81 (0.55–1.61)	0.86 (0.61–1.25)	0.92 (0.66–1.78)	0.93 (0.60–1.45)	0.022
Maximum cIMT (mm)	0.85 (0.60–1.65)	1.00 (0.65–1.30)	1.00 (0.70–1.80)	1.00 (0.65–1.50)	0.021

^a^ *P* values of ANOVA or Kruskal-Wallis test for differences of variables among diabetic nephropathy stage groups.

**Table 2:** Correlation matrix between adiponectin, ox-LDL, and several characteristics of patients with type 2 diabetes in the absence or presence of nephropathy at different stages. Values represent Spearman's correlation coefficients.

	ox-LDL	Age	BMI	Glucose	eGFR	LDL-cholesterol	HDL-cholesterol	Triglycerides
Adiponectin	−0.29^b^	0.26^b^	−0.21^a^	−0.14	−0.33^b^	−0.07	0.09	−0.11
ox-LDL	1	−0.02	0.12	0.05	0.20^a^	0.48^b^	0.08	0.32^b^

^a^Correlation is significant at the 0.05 level.

^b^Correlation is significant at the 0.01 level.

**Table 3:** Regression coefficients (*β*) of multiple regression models with dependent variables ox-LDL or cIMT and independent variables selected based on respective associations.

	*β*	Standard error	*P*
Model for ox-LDL			
Adiponectin	−5.02	2.5	0.049
eGFR	0.12	0.05	0.025
Triglycerides	0.06	0.02	0.004
LDL-cholesterol	0.20	0.05	0.025
Model for cIMT^a^			
eGFR	−0.002	0.001	0.003
Sex	−0.102	0.042	0.017

^a^Age, statin treatment, and HDL-cholesterol were excluded from model.

**Table 4:** Cluster profiles of subgroups of patients with diabetic nephropathy at different stages.

DN stage	Age (y)	cIMT (mm)	Adiponectin (*μ*g/mL)	ox-LDL (U/L)	Cluster			
					1	2	3	4
Controls	64.0 (7.1)	0.83 (0.22)	6.1 (2.9)	67.5 (21.5)				25
1/2	67.2 (8.9)	0.87 (0.18)	4.9 (2.3)	60.4 (17.5)	21	1		
3/4	69.4 (8.1)	0.98 (0.25)	9.0 (8.2)	66.1 (17.4)		42		
5	68.8 (9.3)	0.95 (0.17)	9.5 (6.9)	52.5 (17.2)			23	

Figures represent mean values (standard deviation).

## References

[1] Bojestig M., Arnqvist H. J., Hermansson G., Karlberg B. E., Ludvigsson J. (1994). Declining incidence of nephropathy in insulin-dependent diabetes mellitus. *The New England Journal of Medicine*.

[2] Arora M. K., Singh U. K. (2013). Molecular mechanisms in the pathogenesis of diabetic nephropathy: an update. *Vascular Pharmacology*.

[3] Wada J., Makino H. (2013). Inflammation and the pathogenesis of diabetic nephropathy. *Clinical Science*.

[4] Sharma K., RamachandraRao S., Qiu G. (2008). Adiponectin regulates albuminuria and podocyte function in mice. *The Journal of Clinical Investigation*.

[5] Yilmaz M. I., Saglam M., Qureshi A. R. (2008). Endothelial dysfunction in type-2 diabetics with early diabetic nephropathy is associated with low circulating adiponectin. *Nephrology Dialysis Transplantation*.

[6] Ran J., Xiong X., Liu W. (2010). Increased plasma adiponectin closely associates with vascular endothelial dysfunction in type 2 diabetic patients with diabetic nephropathy. *Diabetes Research and Clinical Practice*.

[7] Miyagishima K., Hiramitsu S., Kato S. (2007). Efficacy of atorvastatin therapy in ischaemic heart disease—effects on oxidized low-density lipoprotein and adiponectin. *Journal of International Medical Research*.

[8] Saito T., Saito O., Kawano T. (2007). Elevation of serum adiponectin and CD146 levels in diabetic nephropathy. *Diabetes Research and Clinical Practice*.

[9] Jorsal A., Tarnow L., Frystyk J. (2008). Serum adiponectin predicts all-cause mortality and end stage renal disease in patients with type I diabetes and diabetic nephropathy. *Kidney International*.

[10] Christou G. A., Kiortsis D. N. (2014). The role of adiponectin in renal physiology and development of albuminuria. *Journal of Endocrinology*.

[11] Sweiss N., Sharma K. (2014). Adiponectin effects on the kidney. *Best Practice and Research: Clinical Endocrinology and Metabolism*.

[12] Zoccali C., Mallamaci F. (2007). Adiponectin and renal disease progression: another epidemiologic conundrum?. *Kidney International*.

[13] Gin H., Rigalleau V., Aparicio M. (2000). Lipids, protein intake, and diabetic nephropathy. *Diabetes & Metabolism*.

[14] Forbes J. M., Coughlan M. T., Cooper M. E. (2008). Oxidative stress as a major culprit in kidney disease in diabetes. *Diabetes*.

[15] Gutwein P., Abdel-Bakky M. S., Doberstein K. (2009). CXCL16 and oxLDL are induced in the onset of diabetic nephropathy. *Journal of Cellular and Molecular Medicine*.

[16] Ujihara N., Sakka Y., Takeda M. (2002). Association between plasma oxidized low-density lipoprotein and diabetic nephropathy. *Diabetes Research and Clinical Practice*.

[17] Ribeiro S., Faria M. D. S., Silva G. (2012). Oxidized low-density lipoprotein and lipoprotein(a) levels in chronic kidney disease patients under hemodialysis: influence of adiponectin and of a polymorphism in the apolipoprotein(a) gene. *Hemodialysis International*.

[18] Wang M., Wang D., Zhang Y., Wang X., Liu Y., Xia M. (2013). Adiponectin increases macrophages cholesterol efflux and suppresses foam cell formation in patients with type 2 diabetes mellitus. *Atherosclerosis*.

[19] Lautamäki R., Rönnemaa T., Huupponen R. (2007). Low serum adiponectin is associated with high circulating oxidized low-density lipoprotein in patients with type 2 diabetes mellitus and coronary artery disease. *Metabolism: Clinical and Experimental*.

[20] Chitalia N., Raja R. B., Bhandara T. (2010). Serum adiponectin and cardiovascular risk in chronic kidney disease and kidney transplantation. *Journal of Nephrology*.

[21] Lisowska A., Musiał W. J., Lisowski P., Knapp M., Małyszko J., Dobrzycki S. (2009). Intima-media thickness is a useful marker of the extent of coronary artery disease in patients with impaired renal function. *Atherosclerosis*.

[22] Hayashi M., Shibata R., Takahashi H. (2011). Association of adiponectin with carotid arteriosclerosis in predialysis chronic kidney disease. *American Journal of Nephrology*.

[23] Lemos M. M., Jancikic A. D. B., Sanches F. M. R. (2010). Intima-media thickness is associated with inflammation and traditional cardiovascular risk factors in non-dialysis-dependent patients with chronic kidney disease. *Nephron—Clinical Practice*.

[24] Karakitsos D., De Groot E., Patrianakos A. P. (2006). Adiponectin and cardiovascular remodeling in end-stage renal disease and co-morbid diabetes mellitus. *American Journal of Nephrology*.

[25] Ignacy W., Chudek J., Adamczak M. (2005). Reciprocal association of plasma adiponectin and serum C-reactive protein concentration in haemodialysis patients with end-stage kidney disease—a follow-up study. *Nephron - Clinical Practice*.

[26] Iordanidou M., Tavridou A., Petridis I. (2010). The serotonin transporter promoter polymorphism (5-HTTLPR) is associated with type 2 diabetes. *Clinica Chimica Acta*.

[27] Parving H. H., Mauer M., Ritz E. (2004). *Diabetic Nephropathy*.

[28] Rossing K., Christensen P. K., Hovind P., Tarnow L., Rossing P., Parving H.-H. (2004). Progression of nephropathy in type 2 diabetic patients. *Kidney International*.

[29] National Kidney Foundation (2002). K/DOQI clinical practice guidelines for chronic kidney disease: evaluation, classification, and stratification. *American Journal of Kidney Diseases*.

[30] Pawlak K., Mysliwiec M., Pawlak D. (2013). Oxidized low-density lipoprotein (oxLDL) plasma levels and oxLDL to LDL ratio—are they real oxidative stress markers in dialyzed patients?. *Life Sciences*.

[31] Mikami S., Hamano T., Fujii N. (2008). Serum osteoprotegerin as a screening tool for coronary artery calcification score in diabetic pre-dialysis patients. *Hypertension Research*.

[32] Levey A. S., Stevens L. A., Schmid C. H. (2009). A new equation to estimate glomerular filtration rate. *Annals of Internal Medicine*.

[33] Vogt W., Nagel D. (1992). Cluster analysis in diagnosis. *Clinical Chemistry*.

[34] Stenvinkel P. (2011). Adiponectin in chronic kidney disease: a complex and context sensitive clinical situation. *Journal of Renal Nutrition*.

[35] Koshimura J., Fujita H., Narita T. (2004). Urinary adiponectin excretion is increased in patients with overt diabetic nephropathy. *Biochemical and Biophysical Research Communications*.

[36] Fujita H., Morii T., Koshimura J. (2006). Possible relationship between adiponectin and renal tubular injury in diabetic nephropathy. *Endocrine Journal*.

[37] Perri A., Vizza D., Lofaro D. (2013). Adiponectin is expressed and secreted by renal tubular epithelial cells. *Journal of Nephrology*.

[38] Levin A., Hemmelgarn B., Culleton B. (2008). Guidelines for the management of chronic kidney disease. *Canadian Medical Association Journal*.

[39] Lipska K. J., Bailey C. J., Inzucchi S. E. (2011). Use of metformin in the setting of mild-to-moderate renal insufficiency. *Diabetes Care*.

[40] Diepeveen S. H. A., Verhoeven G. H. W. E., van der Palen J. (2004). Oxidative stress in patients with end-stage renal disease prior to the start of renal replacement therapy. *Nephron - Clinical Practice*.

[41] Kushiya F., Wada H., Sakakura M. (2003). Effects of lipid abnormalities on arteriosclerosis and hemostatic markers in patients under hemodialysis. *Clinical and Applied Thrombosis/Hemostasis*.

[42] Osorio A., Ortega E., De Haro T., Torres J. M., Sánchez P., Ruiz-Requena E. (2011). Lipid profiles and oxidative stress parameters in male and female hemodialysis patients. *Molecular and Cellular Biochemistry*.

[43] Johnson-Davis K. L., Fernelius C., Eliason N. B., Wilson A., Beddhu S., Roberts W. L. (2011). Blood enzymes and oxidative stress in chronic kidney disease: a cross sectional study. *Annals of Clinical and Laboratory Science*.

[44] Sevinc Ok E., Kircelli F., Asci G. (2012). Neither oxidized nor anti-oxidized low-density lipoprotein level is associated with atherosclerosis or mortality in hemodialysis patients. *Hemodialysis International*.

[45] Gustafsson S., Lind L., Söderberg S., Zilmer M., Hulthe J., Ingelsson E. (2013). Oxidative stress and inflammatory markers in relation to circulating levels of adiponectin. *Obesity*.

[46] Dullaart R. P. F., de Vries R., van Tol A., Sluiter W. J. (2007). Lower plasma adiponectin is a marker of increased intimamedia thickness associated with type 2 diabetes mellitus and with male gender. *European Journal of Endocrinology*.

[47] von Eynatten M., Liu D., Hock C. (2009). Urinary adiponectin excretion: a novel marker for vascular damage in type 2 diabetes. *Diabetes*.

[48] Dursun B., Dursun E., Suleymanlar G. (2009). The effect of hemodialysis on accelerated atherosclerosis in diabetic patients: correlation of carotid artery intima-media thickness with oxidative stress. *Journal of Diabetes and Its Complications*.

